# B cell receptor repertoire reconstitution in patients with neuromyelitis optica spectrum disorder receiving B-cell depletion therapy

**DOI:** 10.3389/fimmu.2025.1673508

**Published:** 2025-10-31

**Authors:** Hyo Jae Kim, Wangyong Shin, Dayoung Seo, Inhye Jang, Jihong Ryu, Lynkyung Choi, Jinhee Kim, Hyunjin Kim, Young-Min Lim, Eun-Jae Lee

**Affiliations:** Department of Neurology, Asan Medical Center, Ulsan University College of Medicine, Seoul, Republic of Korea

**Keywords:** neuromyelitis optica spectrum disorder, B cell receptor sequencing, B cell depletion therapy, rituximab, humoral immunity

## Abstract

**Background:**

Neuromyelitis optica spectrum disorder (NMOSD), driven by AQP4-IgG-producing B cells, is effectively managed with B cell depletion therapy (BCDT), such as rituximab (RTX). Although BCDT may reset the B cell compartment, its effects on B cell receptor (BCR) repertoire, clonality, isotype distribution, and somatic hypermutation (SHM) remain poorly understood. To examine how BCDT alters BCR features by comparing BCR repertoires between patients with NMOSD treated with RTX and azathioprine (AZA).

**Methods:**

From a prospective cohort, we recruited patients with NMOSD, including those on AZA (n = 11) and those 6–12 months post-RTX treatment (n = 9). Immunoglobulin heavy-chain libraries were generated from peripheral blood mononuclear cells and sequenced using Illumina MiSeq (2 × 300 bp). BCR features analyzed included isotype frequencies, D50 diversity index, top 10% clone fraction, SHM rates, and IGHV and IGHJ gene usage.

**Results:**

Age (median 50 years) and disability scores were similar between the groups. In the RTX group, the median time since the last infusion was 9 months. RTX treatment led to a naïve B cell-dominant profile, with significantly reduced IgG1–IgG4 levels and unchanged IgA levels. Clonality was reduced, especially within the IgG isotype. SHM frequency was similar between groups, with no significant differences observed across individual isotypes. RTX also resulted in marked depletion of IGHV3-23, IGHV3-11, and IGHV3-73, along with IgG subclass-specific reductions in IGHV1-18, IGHV1-3, IGHV1-46, IGHV1-69, and IGHJ4.

**Conclusion:**

Six to twelve months after RTX treatment, patients with NMOSD display a rejuvenated BCR repertoire characterized by naïve B cell predominance, reduced class-switched clonality, and selective loss of V gene segments. These findings support the mechanism by which BCDT resets pathogenic memory B cells and offer benchmarks for monitoring B cell reconstitution in NMOSD.

## 1 Introduction

Neuromyelitis optica spectrum disorder (NMOSD) is a chronic inflammatory disease of the central nervous system characterized by recurrent episodes of optic neuritis and longitudinally extensive transverse myelitis (1). Its pathogenesis is driven by humoral autoimmunity, notably the production of pathogenic IgG autoantibodies targeting aquaporin-4 (AQP4) on astrocytes (2, 3). B cells and plasmablasts play a central role in NMOSD immunopathology through the production of AQP4-IgG and the secretion of pro-inflammatory cytokines (4, 5). Elevated circulating plasmablast levels in patients reflect ongoing B cell activation, providing a strong rationale for B cell-targeted therapies (6).

B cell depletion therapies (BCDTs), including rituximab (RTX) and inebilizumab, have demonstrated robust efficacy in NMOSD, significantly reducing relapse rates (7, 8). Beyond antibody depletion, BCDT appears to reset the B cell compartment (9). Circulating B cells are markedly reduced for several months post-treatment, followed by reconstitution initiating around 6 months and extending to 12 months (10). BCDT has also been associated with increased frequencies of regulatory B cells during reconstitution (11). This immunological reset may result from the selective depletion of pathogenic memory clones and the preferential re-emergence of a less autoreactive, more tolerogenic B cell pool. Conversely, conventional immunosuppressants such as azathioprine (AZA) or mycophenolate exert continuous, non-selective immunosuppressive pressure without complete B cell depletion, potentially allowing the persistence of long-lived memory clones and autoreactive subsets (12, 13). Direct B cell receptor (BCR) repertoire analysis provides critical insights into the extent of B cell remodeling (14). Nevertheless, whether BCDTs modify the composition and diversity of the BCR repertoire remains to be elucidated.

We hypothesized that BCDTs, by inducing B cell depletion followed by naïve-skewed reconstitution, would generate a more diverse, less mutated BCR repertoire enriched for IgM and reduced class-switched IgG clones, in contrast to the repertoire maintained under AZA treatment. To test this hypothesis, we conducted a cross-sectional analysis of peripheral blood BCR sequences from patients with NMOSD, receiving either RTX or AZA. We also evaluated whether BCDTs mitigate disease-associated biases in immunoglobulin heavy-chain variable (IGHV) and immunoglobulin heavy-chain joining (IGHJ) gene usage. Our results showed that RTX induced a marked shift toward a naïve-dominated, low-clonality B cell profile, including substantial depletion of class-switched IgG clones and a distinct pattern of IGHV usage. Conversely, AZA-treated patients exhibited features of a chronically stimulated repertoire, including expanded IgG clones. Collectively, these findings suggest that BCDT reshapes the B cell landscape and contributes to immune rebalancing in NMOSD.

## 2 Materials and methods

### 2.1 Study cohort

This study analyzed blood samples and clinical data collected between 2021 and 2022 from a prospective NMOSD cohort established in 2018 at the Department of Neurology, Asan Medical Center (Seoul, Korea). NMOSD diagnosis was based on the 2015 criteria (1), and anti-AQP4-IgG seropositivity was confirmed using a cell-based assay (15, 16). We included patients who were in remission for a minimum of 6 months after the last clinical relapse and were receiving maintenance therapy with either RTX or AZA. Patients with a history of cancer, active or chronic infection, other autoimmune or chronic inflammatory diseases, or use of immunosuppressive agents other than RTX or AZA within the previous 12 months were excluded.

RTX induction therapy consisted of two 1,000 mg infusions administered two weeks apart, followed by 1,000 mg maintenance infusions when CD19^+^ B cells exceeded 1% or CD19^+^CD27^+^ memory B cells exceeded 0.05% of peripheral blood mononuclear cells (PBMCs) (17). AZA was administered at a stable dose of 2–3 mg/kg/day for at least 6 months. We stratified patients to assess the impact of BCDT on the BCR repertoire: specifically, an RTX group sampled at least 6 months after the last RTX infusion and a comparator group receiving AZA monotherapy, which is not expected to directly reshape the BCR repertoire.

Baseline demographic and clinical variables (age, age at onset, sex, comorbidities, and concomitant medications) were obtained from the cohort registry. Expanded Disability Status Scale (EDSS) scores (18) were assessed by a board-certified neurologist at the time of sampling. We also recorded the cumulative number of demyelinating relapses affecting the brain, spinal cord, and optic nerve. Then, the disease duration was defined as the interval between symptom onset and sampling. The study was approved by the Institutional Review Board (IRB) of the Asan Medical Center (No. 2018-0653), and all participants provided written informed consent.

### 2.2 Sample collection and BCR sequencing

PBMCs were isolated from whole blood using density gradient centrifugation. PBMCs were processed to generate template-switched cDNA from total RNA, and immunoglobulin heavy-chain (IGH) libraries were prepared for high-throughput sequencing. IGH cDNA was produced using a template-switching reverse transcription protocol that appends a non-templated 5′ GGG to full-length transcripts, yielding a 5′-GGG–leader–V–(D)–J–constant–3′ architecture. From this cDNA (since no unique molecular identifier (UMI) was incorporated), IGH amplicons were generated using semi-nested, multiplex PCR designed to capture the complete rearranged V(D)J while encoding isotype/subclass information. Forward primers targeted the IGH leader or framework region 1 across IGHV families, and reverse primers annealed within the constant region at sites ~20–23 bp 3′ to the VDJ–constant junction, enabling discrimination of IgM, IgD, IgE, IgG1–4, and IgA1–2. Libraries were sequenced on an Illumina MiSeq (2 × 300 bp run configuration). Consistent with the template-switching layout, Read 2 typically begins at the cDNA 5′-end with the GGG motif, followed by the leader and the full V(D)J segment, whereas Read 1 initiates from the constant-region primer and aligns in reverse orientation. Healthy-donor PBMC libraries were sequenced alongside study samples to monitor amplification balance and run performance.

### 2.3 Sequence preprocessing and clonal assignment

Raw sequencing reads underwent quality control and processing using the Immcantation framework. First, we used pRESTO for read assembly and quality filtering: paired-end reads were aligned and merged, and sequences with low-quality bases or frameshifts were removed (19). The UMI consensus assembly was not applicable, since our protocol did not utilize UMIs. We then used IgBlast (NCBI, v1.17) to align each sequence to immunoglobulin germline gene databases (the IMGT reference for IGHV, IGHD, and IGHJ gene segments) (20, 21). Sequences were annotated with the best-matched germline V, D, and J genes and the location of the junction (specifically the CDR3). We included only productive, full-length IGH rearrangements in the final dataset (i.e., sequences with in-frame VDJ and no stop codons). The isotype (IGHM, IGHD, IGHG1–4, and IGHA1–2) for each sequence was determined via matching to constant region primers or sequence tags in the read. Clonal clustering was performed using Change-O (Immcantation suite). Sequences were grouped into clones if they shared the same IGHV and IGHJ gene usage and had highly similar CDR3 sequences (defined by a lineage clustering threshold of 85% CDR3 nucleotide identity, allowing for sequencing error and somatic mutation) (22). Each clone was thus intended to represent a unique B cell lineage derived from a common naïve B cell precursor.

### 2.4 BCR repertoire analysis

For each patient’s repertoire, we computed summary metrics. Isotype usage: We calculated the proportion of sequences belonging to each isotype (e.g., % IGHM and % IGHG1) relative to the total IGH sequences for that patient. Clonality and diversity: We assessed clonality using two complementary measures: (1) D50 index (D50), which is defined as the minimum cumulative fraction of clones that accounts for 50% of the total sequencing reads (23). A low D50 indicates that relatively few clones contribute half of all BCR sequences (i.e., high clonality or oligoclonality), whereas a higher D50 reflects a more even distribution of sequence counts across clones (i.e., greater diversity). We computed D50 for the overall repertoire of each patient, and additionally within subsets of sequences stratified by isotype (e.g., considering only IGHG sequences). (2) Top 10% clone fraction: We determined the fraction of each patient’s total sequences contributed by the most abundant 10% of clones. This metric captures clonal dominance; a higher value indicates that the top clones account for a large portion of the repertoire, reflecting skewed clonality. Somatic hypermutation (SHM) analysis: Using Change-O and SHazaM (Immcantation suite tools) (22), we inferred the germline IGHV sequence for each clone and calculated the somatic mutation frequency as the number of nucleotide mismatches in the V region divided by the V region length. We obtained per-sequence and per-clone mutation frequencies and summarized each patient’s repertoire using the mean mutation frequency of all sequences (overall SHM level). We also examined SHM stratified by isotype, since IGHM and IGHD sequences (largely naïve or IgM-memory B cells) are expected to have low SHM, whereas IGHG and IGHA sequences (from class-switched memory B cells) typically harbor higher mutation levels. V and J gene usage: We compiled the frequency of each IGHV gene and each IGHJ gene among productive sequences per patient. For group-level comparison, we expressed gene usage frequencies as a percentage of total sequences (or of total sequences within a subset, such as IGHG sequences) for that patient and then compared the values between the AZA and RTX groups.

### 2.5 Statistical analysis

The isotype proportion, clonality, SHM, and IGHV and IGHJ usage proportions were measured in this study. Mann–Whitney U tests were used to compare numerical data. All tests were two-sided, and a *p*-value of < 0.05 was considered statistically significant. All statistical analyses were performed using Python (version 3.9.1) or R (version 4.1.0). Data are presented as the mean ± standard error of the mean (SEM) unless otherwise specified.

## 3 Results

### 3.1 Patient characteristics

During the study period, a total of 20 patients (9 RTX and 11 AZA) met the inclusion criteria and were included in the PBMC analysis (**Table 1**). The median ages of the RTX and AZA groups were 49 and 57 years, respectively, with no statistically significant difference. Both groups exhibited a female predominance (88.9% vs. 90.9%, *p* > 0.999). Disease duration and EDSS scores were comparable between the groups. At the time of sampling, patients treated with RTX had a median of 9 months from their last infusion and 36 months from RTX initiation, whereas patients treated with AZA had a median of 42 months from treatment initiation. In both groups, no concomitant immunosuppressive agents were used beyond RTX or AZA. According to the inclusion criteria, no patient in either group had experienced a clinical NMOSD relapse within 6 months prior to sampling. Baseline annualized relapse rates were higher in the RTX group compared to those in the AZA group (0.39 vs. 0.29, *p* < 0.020).

**Table 1:** Patient’s characteristics.

Variables	Rituximab (n = 9)	Azathioprine (n = 11)	*p*-value
Female (%)	8 (88.9)	10 (90.9)	> 0.999
Age, median (IQR)	49 (44–51)	57 (48–68)	0.305
Disease duration, years, median (IQR)	16 (7–19)	6 (4–12)	0.137
EDSS, median (IQR)	3 (2–6)	3 (2–4.3)	0.730
ARR, median (IQR)	0.39 (0.29–0.08)	0.29 (0.17–0.49)	0.020
Last rituximab-to-sample interval, months, median (IQR)	9 (7–12)	NA	NA
Initial rituximab-to-sample interval, months, median (IQR)	36 (22–41)	NA	NA
Initial azathioprine-to-sample interval, months, median (IQR)	NA	42 (31–114)	NA

IQR, interquartile range; EDSS, Expanded Disability Status Scale; ARR, Annualized Relapse Rate.

### 3.2 Isotype distribution in AZA vs RTX repertoires

**Figure 1:**
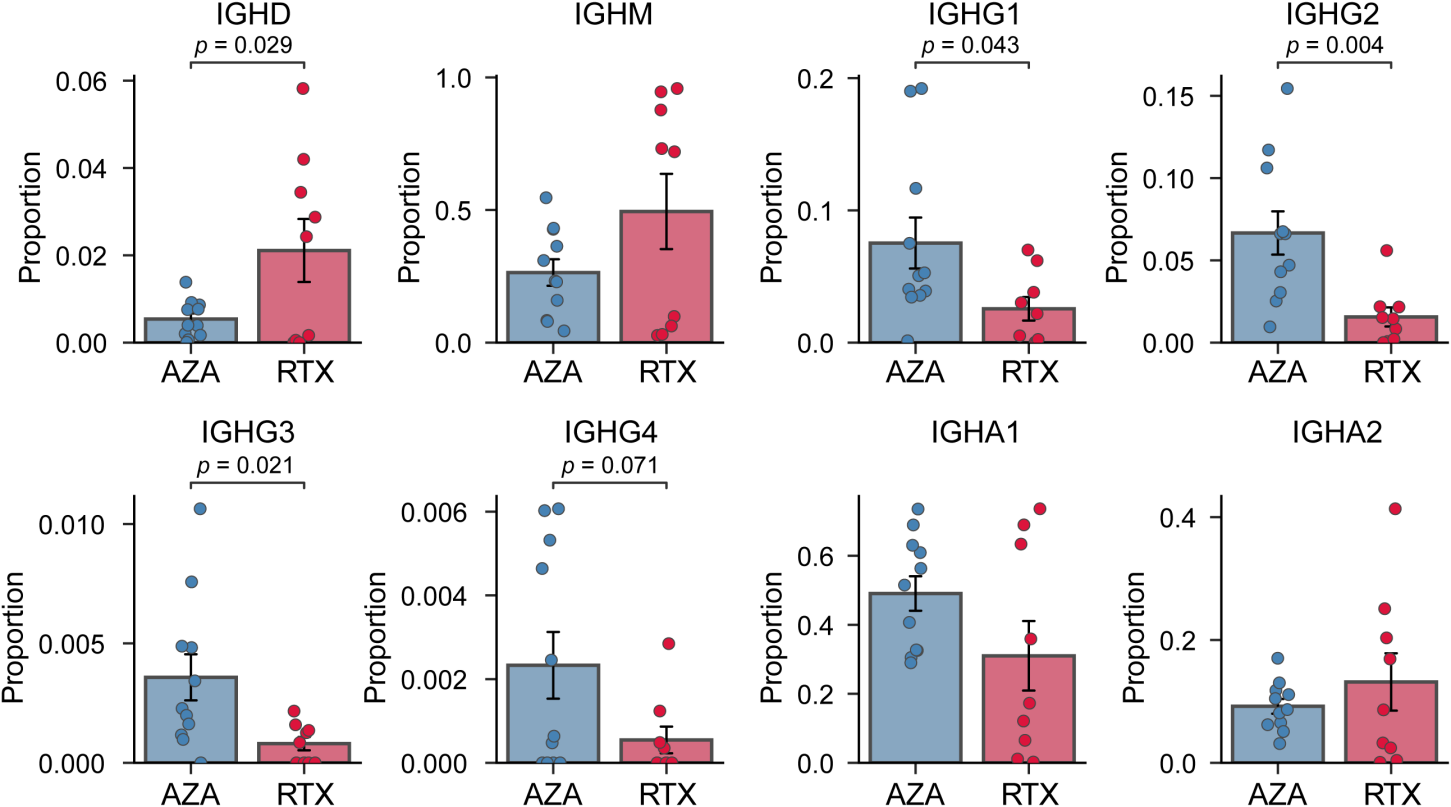
Comparison of immunoglobulin heavy-chain isotype proportion. Proportion of BCR sequences for each isotype (IGHD, IGHM, IGHG1, IGHG2, IGHG3, IGHG4, IGHA1, and IGHA2) in the azathioprine- (AZA) and rituximab- (RTX) treated groups. Each point represents an individual patient sample; bars indicate group means, and error bars denote standard error of the mean.

As hypothesized, we observed marked differences in the composition of immunoglobulin isotypes between the two groups (**Figure 1**). In patients treated with AZA, class-switched isotypes constituted a large fraction of the circulating BCR repertoire, whereas patients treated with RTX showed a bias toward unswitched isotypes (IGHD, IGHM, generally expressed by naïve or memory B cells). The IGHD isotype was significantly elevated in the RTX group compared to the AZA group (2.1% vs. 0.5%, *p* = 0.029), indicative of the re-emergence of naïve B cells co-expressing IGHM and IGHD. Within the RTX group, four patients exhibited markedly low IGHD proportions. However, the IGHD proportion was not significantly associated with the interval from the last RTX infusion to sampling or with disease duration (**Supplementary Figure S1**). This finding suggests that these outliers reflect biological variability in the kinetics of B cell reconstitution rather than effects of treatment timing or disease chronicity. The IGHM isotype also tended to be higher in the patients of the RTX group (49.5% vs. 26.4%, *p* = 0.115), although this did not reach statistical significance given the observed variability. IGHG subclasses (IGHG1, IGHG2, and IGHG3) were significantly underrepresented in the patients treated with RTX. In the RTX group, IgG1 averaged only 2.6% compared to 7.5% in the AZA group. The differences for each subclass were statistically significant (*p* = 0.043 for IgG1; *p* = 0.004 for IgG2; *p* = 0.021 for IgG3). IGHA subtypes proportions showed no significant differences between the groups. This suggests that IGHA-expressing B cells (often originating from mucosal immune responses) were not preferentially depleted or expanded in either treatment. Overall, these results demonstrate that patients with NMOSD treated with RTX, at 6–12 months post-therapy, have a BCR isotype profile shifted toward IgM (naïve B cells) with a paucity of IgG sequences. Conversely, patients treated with AZA maintain higher levels of IgG class-switched B cells. The preservation of IgA in the RTX group indicates that not all class-switched compartments are equally affected.

### 3.3 Clonality and diversity

**Figure 2:**
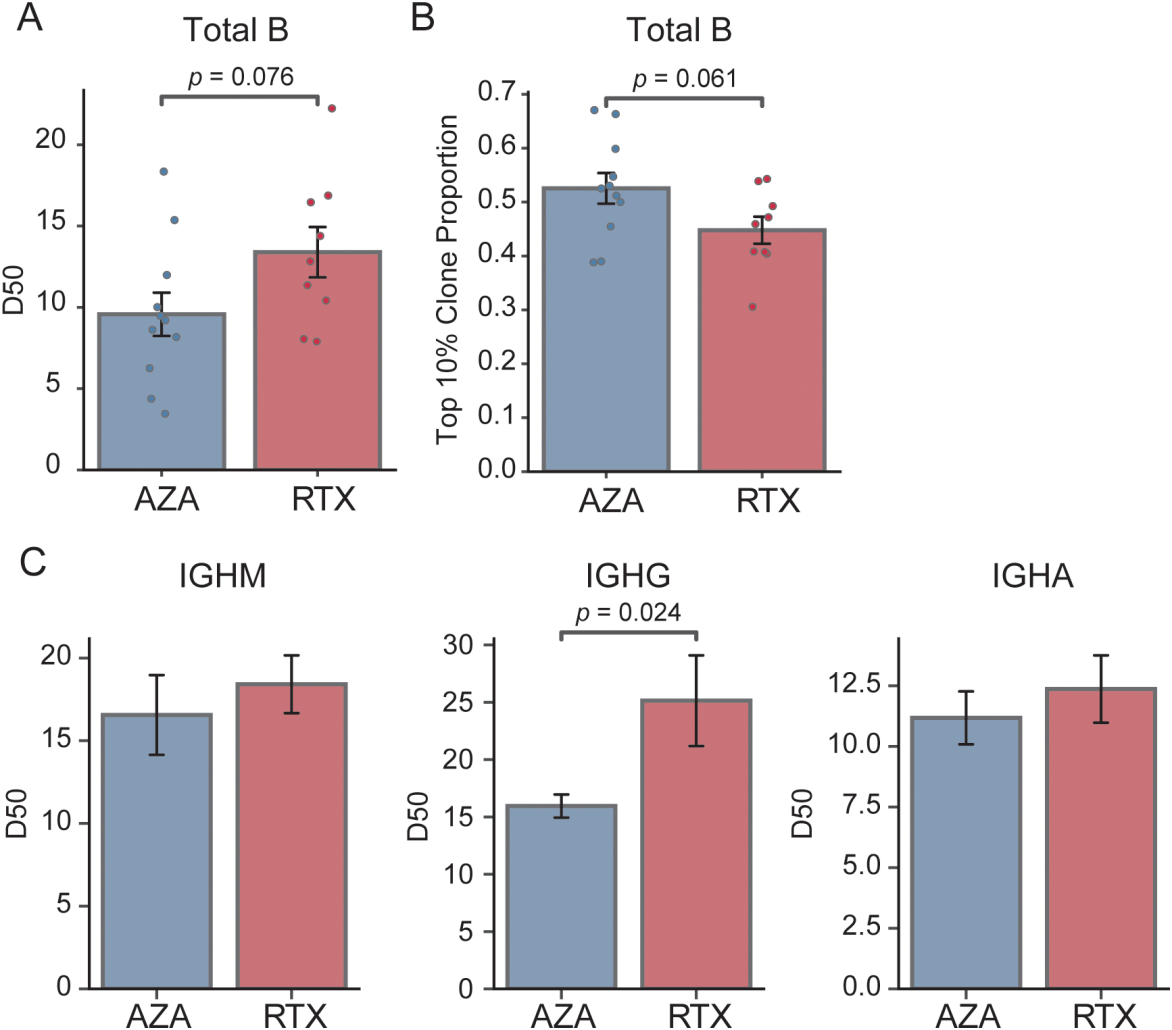
Comparison of clonality and diversity of the BCR repertoire. **(A)** Comparison of the D50 diversity index calculated from total B cell repertoires. Each point represents an individual patient sample; bars indicate group means, and error bars denote standard error of the mean. **(B)** Proportion of sequences accounted for by the top 10% most abundant clones in each treatment group; bars show group means and error bars represent standard error of the mean. **(C)** D50 diversity index stratified by isotype (IGHM, IGHG, and IGHA) for the azathioprine- (AZA) and rituximab- (RTX) treated groups; bars depict mean values and error bars correspond to standard error of the mean.

We next compared the clonal composition of BCR sequences between the two groups. We reasoned that RTX, by resetting the B cell pool, might produce a more diverse, evenly distributed repertoire (lower clonality) relative to AZA. We quantified diversity and clonality using the D50 metric and the top 10% clone fraction, as described in the Methods. **Figure 2A** shows the D50 index for each group: on average, the RTX group tended to have a higher D50 than that of the AZA group (0.13 vs. 0.10, *p* = 0.076), suggesting a more diverse BCR repertoire and lower clonality in the RTX group. In parallel, the top 10% clone fraction was lower in the patients in the RTX group (**Figure 2B**): the most abundant 10% of clones accounted for approximately 44.8% of total sequences in the RTX group, compared to 52.6% in the AZA group. This again points to lower dominance by top clones in patients treated with RTX (*p* = 0.06 for group difference). Together, these two metrics consistently show that the B cell repertoire after RTX treatment tends to be more polyclonal compared to that under AZA therapy.

We further analyzed clonality within individual BCR isotype subsets to determine whether specific compartments contributed to these differences (**Figure 2C**). There was no significant difference in the IgM D50 between the RTX and AZA groups (0.18 vs. 0.17, *p* = 0.557), indicating comparable diversity within the naïve B cell pool. Conversely, the IgG-bearing repertoire showed a pronounced difference: patients treated with RTX had a significantly higher IgG D50 than those receiving AZA (0.25 vs. 0.16, *p* = 0.024). This indicates a greater clonal diversity in the IgG compartment after RTX treatment. Specifically, although a small number of IgG clones dominated the repertoire in the patients treated with AZA, consistent with clonal expansion of memory B cells or plasmablasts, the patients treated with RTX exhibited a more evenly distributed IgG repertoire, with no single predominant clone. These results suggest that the class-switched memory compartment in the patients treated with RTX is not only reduced in size, as indicated by isotype frequencies, but also exhibits lower clonal skewing. This likely reflects the depletion of previously expanded clones following B cell depletion. Conversely, the diversity of IgA sequences did not differ significantly between the groups, consistent with earlier observations that the proportion of IgA isotypes remains unaffected by RTX treatment (24).

### 3.4 Somatic hypermutation

**Figure 3:**
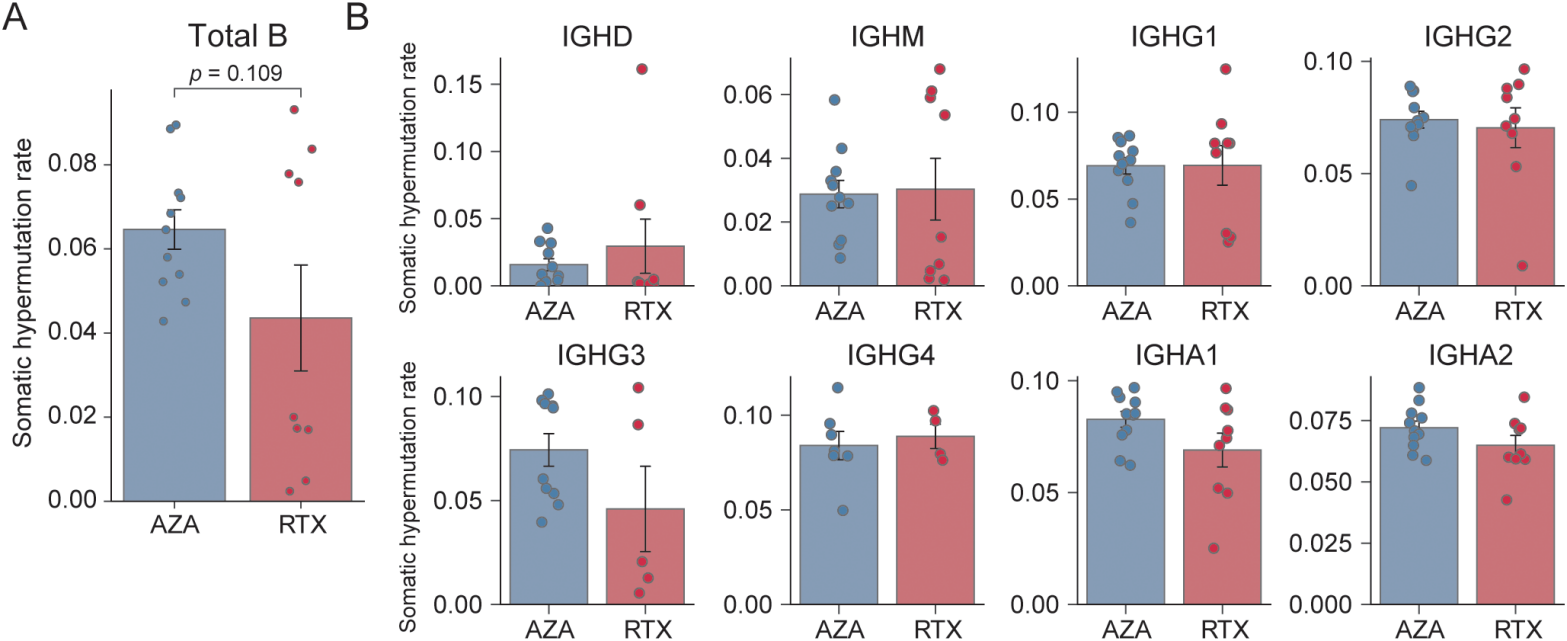
Somatic hypermutation frequency. **(A)** Overall mean somatic hypermutation (SHM) frequency across all IGH sequences. Each point denotes a single sample; bars represent group means, and error bars indicate standard error of the mean. **(B)** Mean SHM frequency stratified by isotype (IGHD, IGHM, IGHG1, IGHG2, IGHG3, IGHG4, IGHA1, and IGHA2) for the azathioprine- (AZA) and rituximab- (RTX) treated groups; bars depict means, and error bars correspond to standard error of the mean.

SHM in immunoglobulin genes reflects an antigen-driven selection and is characteristic of germinal center-experienced B cells, such as class-switched memory B cells and plasmablasts. Upon analyzing all immunoglobulin sequences collectively, both treatments showed comparable SHM levels (**Figure 3A**), with the mean nucleotide mutation frequency being 4.4% in the RTX group compared to 6.5% in the AZA group (*p* = 0.109). Notably, SHM rates in the RTX group appeared to cluster into two distinct subsets: a higher group (> 7.5%) and a lower group (< 2.0%). We further examined SHM in an isotype-specific manner, as the overall mutation frequency may be influenced by isotype composition. No statistically significant differences in SHM were observed between the treatment groups for IgG1, IgG2, IgG3, or IgG4, with comparable mutation burdens across all subclasses (**Figure 3B**). Notably, although the patients treated with RTX had substantially fewer IgG sequences (**Figure 1**), the remaining sequences exhibited comparable levels of SHM to those in the AZA group. Similarly, SHM frequencies in IgA1 and IgA2 did not differ significantly between the groups. These findings suggest that RTX primarily shifts the repertoire toward a higher proportion of naïve B cells, rather than resetting the mutation burden of memory B cells that re-emerge after depletion.

### 3.5 IGHV and IGHJ gene segment usage

We examined whether immunoglobulin heavy chain variable (IGHV) and joining (IGHJ) gene usage differed between the AZA- and RTX-treated groups. Certain IGHV genes have been associated with autoreactivity and clonal expansion in autoimmune diseases (25). We specifically investigated whether RTX preferentially depleted clones using IGHV segments implicated in disease or characteristic of memory B cell responses.

**Figure 4:**
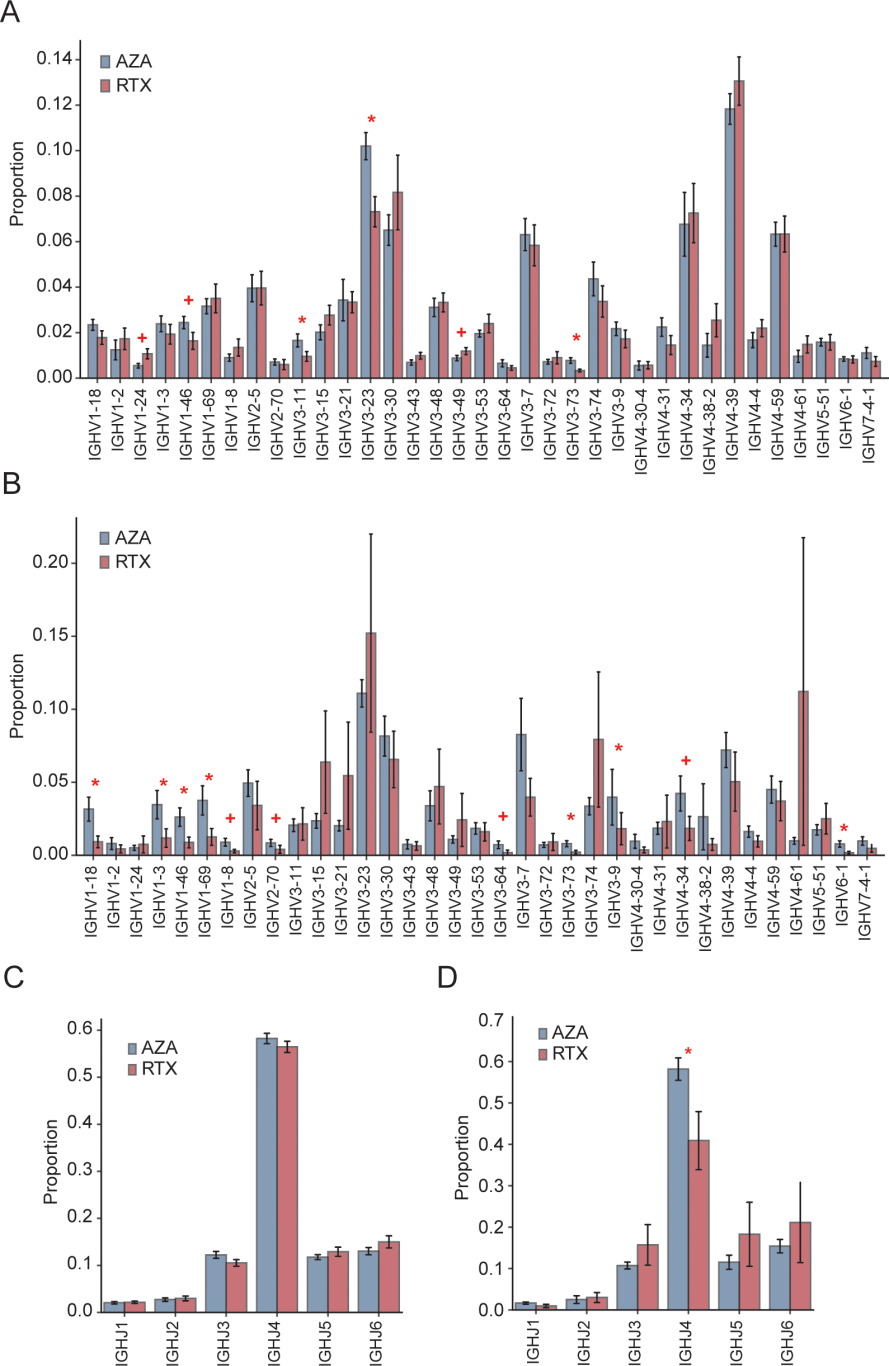
IGHV and IGHJ gene usage. **(A)** Overall frequency of IGHV gene segments among total IGH sequences in the azathioprine- (AZA) and rituximab- (RTX) treated groups; bars and error bars indicate group mean ± standard error of the mean. **(C)** Overall distribution of the IGHJ gene segments across all IGH sequences in the AZA- and RTX-treated groups; bars and error bars indicate mean ± standard error of the mean. **(B)** IGHV usage within the IgG subset only in the AZA- and RTX-treated groups; bars mean ± SEM. **(D)** IGHJ usage within the IgG subset only in the AZA- and RTX-treated groups; bars mean ± standard error of the mean. * Indicates *p* ≤ 0.05; + indicates 0.05 < *p* ≤ 0.10.

IGHV usage patterns showed both overlapping and distinct features between the groups (**Figure 4A**). In both AZA and RTX groups, IGHV4–39 was the most frequently used gene. However, three IGHV3-family genes (IGHV3-23, IGHV3-11, and IGHV3-73) were significantly underrepresented in patients treated with RTX, suggesting that these clones were either selectively depleted or less likely to reconstitute after RTX treatment. To specifically assess class-switched IgG-secreting B cells, IGHV gene usage was further analyzed within IgG sequences (**Figure 4B**). Group differences became more pronounced, particularly for the IGHV1 family. Four IGHV1 genes (IGHV1-18, IGHV1-3, IGHV1-46, and IGHV1-69) were significantly less frequent in the RTX IgG repertoire compared to that in the AZA group. These genes collectively accounted for a substantial proportion of the IgG clones in the patients treated with AZA; for example, IGHV1–69 comprised 3.8% of IgG sequences in the AZA group but only 1.2% in the RTX group. This suggests that RTX selectively eliminates or impairs the reconstitution of IgG memory B cells using the IGHV1-family genes. Additionally, IGHV3-73, IGHV3-9, and IGHV4–34 were also reduced in the IgG sequences of the RTX-treated group.

Regarding the IGHJ gene usage (**Figure 4C**), the overall distribution of IGHJ1–IGHJ6 segments did not differ significantly between the groups when all the sequences were analyzed, with IGHJ4 being the most prevalent in both. This indicates that the RTX treatment does not broadly alter the J gene usage at the repertoire level. However, when focusing on IgG sequences (**Figure 4D**), the IGHJ4 usage was significantly lower in the patients treated with RTX, likely reflecting the depletion of the dominant IgG clones that preferentially use this segment.

## 4 Discussion

In this study, we conducted a detailed comparison of peripheral BCR repertoires in patients with NMOSD treated with AZA versus those receiving RTX 6–12 months post-therapy. Our findings reveal that B cell depletion with RTX leads to a profound remodeling of the BCR repertoire relative to conventional immunosuppression with AZA. We observed that B cells of patients treated with RTX are skewed toward naïve phenotypes and exhibit reduced clonality, particularly among class-switched IgG B cells. Conversely, patients treated with AZA maintain the hallmarks of an antigen-experienced, chronically stimulated B cell repertoire, including abundant IgG clones, higher clonality, and usage of specific IGHV genes. These findings provide insights into the distinct immunological landscapes shaped by RTX and AZA, and they offer a potential mechanism of how RTX may achieve more effective disease control in NMOSD.

A key distinction was the marked depletion of IgG-expressing B cell clones in patients treated with RTX. Although some B cell reconstitution occurred 6 to 12 months after RTX, IgG sequences remained a small minority and showed high diversity without dominant clonal expansions. This suggests that RTX preferentially eliminates or delays the return of class-switched memory B cells, which likely include autoreactive clones such as those targeting AQP4. Conversely, patients treated with AZA retained substantial IgG populations, consistent with the persistence of memory B cells (e.g., long-lived memory B cells) and plasmablasts, since AZA suppresses immune activity without depleting B cells. The high clonality and skewed IGHV usage in the AZA group support the continued expansion of potentially pathogenic clones. This pattern aligns with prior reports of oligoclonal B cell expansions in patients with NMOSD without B cell depletion (14, 26). In our cohort, the AZA group reflected this profile, whereas the RTX-treated patients showed greater repertoire diversity and absence of dominant clones, suggesting an effective clonal resetting by RTX.

The particular effect of RTX on the IgG^+^ clones can be understood by its mechanism of action. RTX targets CD20, which is expressed on pre-B, naïve, and memory B cells, but not on plasma cells (7, 27). Class-switched memory B cells, including IgG^+^ and IgA^+^ subsets, express CD20 and are therefore directly depleted by RTX (27). Although naïve B cells are also targeted, they are continuously regenerated from the bone marrow and tend to repopulate earlier than memory cells (28). Consequently, the B cell pool post-RTX is predominantly composed of transitional and naïve cells, with memory B cells remaining scarce for an extended period (10). Our findings are consistent with this pattern. At 6 to 12 months post-treatment, the B cell repertoire in the patients treated with RTX is largely naïve-derived (reflected by a high proportion of IgM^+^ cells), whereas IgG^+^ clones remain infrequent and diverse. Memory B cells that reappear early may represent clones that escaped depletion, possibly by residing in protective niches or downregulating CD20 expression (29). These are likely to be atypical or less pathogenic, whereas highly expanded, autoreactive clones may have been preferentially eliminated, possibly due to greater CD20 expression or increased circulation, which could have made them more “accessible” to RTX. Conversely, some naïve B cells in the bone marrow may partially evade initial targeting. Overall, this results in a selective depletion of the antigen-experienced B cell repertoire.

Our IGHV gene analysis further supports that RTX-mediated depletion was not random but selectively affected specific V gene–defined clones. Notably, IGHV3-23, IGHV3-11, and IGHV3–73 were significantly reduced in the overall repertoire of the patients treated with RTX. These IGHV3 genes have been previously associated with clonal expansions in NMOSD and related autoimmune conditions (14), implying their involvement in common antigenic responses, potentially including AQP4. RTX appeared to eliminate the overrepresentation of these genes, normalizing their usage toward levels seen in naïve repertoires. Within the IgG compartment, we observed a marked reduction in IGHV1-69, IGHV1-46, IGHV1-18, and IGHV1–3 in the patients treated with RTX. Their absence likely reflects the depletion of long-lived memory B cell clones accumulated over time. In effect, RTX reboots the B cell repertoire, requiring regeneration from naïve precursors. As a result, the post-RTX repertoire lacks previously expanded clones and certain immunodominant V gene usages characteristic of the pre-treatment memory pool. This may be advantageous in autoimmune settings, where pathogenic clones are among those eliminated.

An interesting observation was the relative preservation of the IgA-expressing B cells in the patients treated with RTX, both in frequency and diversity. One possibility is that IgA-producing B cells often reside in mucosal tissues, such as gut-associated lymphoid tissue, and circulate less extensively (24). Some may also be long-lived plasma cells in the bone marrow or mucosa, which do not express CD20 and are therefore unaffected by RTX. Furthermore, mucosal IgA responses may regenerate more rapidly after B cell depletion, driven by continuous exposure to commensal antigens (30). By 6 to 12 months post-RTX, the IgA repertoire in blood closely resembled that of patients treated with AZA, suggesting that any depletion of IgA memory B cells was either transient or incomplete. This highlights a mechanistic distinction: RTX primarily targets systemic IgG memory B cells, whereas IgA-mediated immunity remains largely intact or recovers quickly. Clinically, this may be beneficial, as it preserves mucosal immune defenses (e.g., against infections) while suppressing pathogenic IgG clones. Alternatively, this pattern may reflect disease specificity, given that NMOSD is primarily driven by AQP4-IgG1 autoantibodies (31). The minimal effect on IgA clones suggests that RTX selectively eliminates B cells that are most relevant to disease pathogenesis.

Although we anticipated a reduction in overall SHM frequency after RTX treatment due to the influx of unmutated naïve B cells, no significant difference was observed between the RTX and AZA groups. SHM rates in the RTX cohort instead segregated into two clusters, representing highly and minimally mutated sequences. This suggests that while naïve B cells dilute the average SHM level, the memory B cells that repopulate after RTX treatment remain highly mutated. Isotype-specific analysis confirmed that within IgG and IgA subsets, SHM burdens were comparable between the RTX and AZA groups. This indicates that RTX does not revert memory B cells to a less mutated state but instead removes most memory cells, leaving a small, residual memory compartment that retains its mutational history. In the context of NMOSD, it is desirable that the pathogenic antigen, AQP4, is not frequently encountered. However, if antigen exposure does occur, there is a theoretical risk that newly generated B cell clones could develop autoreactivity. Our data cannot determine whether the newly emerging memory clones in the patients treated with RTX are directed against autoantigens or benign antigens. However, the absence of clinical relapses at the time of sampling suggests that truly pathogenic AQP4-specific clones may not have re-emerged, and that the existing memory B cells might instead be reactive against non-pathogenic antigens.

The observed differences may have important clinical implications. Our findings indicate that RTX influences NMOSD disease activity not only through B-cell depletion, but also by reshaping B cell phenotypes. Although B cells begin to repopulate 6 to 12 months after RTX treatment, the majority exhibit naïve features rather than pathogenic profiles. This suggests that simply tracking total B cell counts may not accurately reflect relapse risk. Currently, clinicians monitor memory B cells or IgG^+^ B cells to guide retreatment (32), but this approach often fails to predict relapses reliably (17). Our findings indicate that evaluating BCR-repertoire clonality or tracking the re-emergence of specific expanded clones could refine treatment decisions. A polyclonal, naïve-like repertoire may indicate sustained remission, whereas a highly clonal, skewed repertoire could signal the return of pathogenic B cells. Further studies are warranted to validate this approach as a tool for relapse risk stratification in NMOSD.

Several limitations of our study should be acknowledged. The sample size was modest, reflecting the challenge of obtaining peripheral blood samples from well-characterized patients with NMOSD undergoing specific treatments. As a result, some observed trends may not have reached statistical significance. Our analysis focused solely on peripheral blood B cells, whereas disease-relevant B cell activity in NMOSD may also occur in tissues such as the cerebrospinal fluid or secondary lymphoid organs (33), which were not assessed. Moreover, we did not perform flow cytometry-based subset analysis, which is the conventional method to directly quantify naïve and memory B cell populations. Instead, naïve predominance was inferred from repertoire features, specifically the reduction in class-switched IgG memory clones, decreased clonality, and selective loss of memory-associated IGHV and IGHJ genes. Future confirmatory studies incorporating direct B cell subset analysis will be valuable to validate and extend our repertoire-based findings. Although we attribute the observed differences to treatment effects, potential baseline differences between patient groups must also be considered. Even though patients were matched and no major disparities were identified, unmeasured variables such as prior infections or vaccination history could influence B cell repertoires independently of therapy (34, 35). Furthermore, we were not able to stratify analyses by disease status (e.g., remission vs. relapse) or by treatment-naïve versus previously treated patients, which further limits the granularity of our findings. A longitudinal design, sampling patients before and after RTX treatment or following a switch from AZA to RTX, would more directly assess treatment-induced changes. Conversely, our cross-sectional approach relies on between-group comparisons. Despite these limitations, the observed differences were substantial and biologically plausible, supporting the relevance of our findings.

## 5 Conclusions

In conclusion, our study shows that RTX therapy in NMOSD not only suppresses disease activity but also profoundly reshapes the B cell immune repertoire in the months after treatment. Patients treated with RTX exhibit a rejuvenated profile dominated by naïve B cells and lacking many of the expanded, class-switched clones observed under AZA treatment. These findings support the concept of a BCDT-induced “immune reset,” which likely contributes to its superior clinical efficacy. Pathogenic IgG^+^ memory B cell clones appear particularly susceptible to BCDT, as reflected by their marked reduction or absence post-treatment. Future research should focus on characterizing the antigen specificities of B cells that re-emerge after BCDT and determining their association with relapse risk in NMOSD and related disorders.

## Data Availability

The data presented in this study are deposited in the Zenodo repository under the accession number 17360431.
