# Supplementary material for: B cell receptor repertoire reconstitution in patients with neuromyelitis optica spectrum disorder receiving B-cell depletion therapy
Source: Front Immunol. 2025 Oct 31;16:1673508. doi: 10.3389/fimmu.2025.1673508 (PMC12615372; doi:10.3389/fimmu.2025.1673508)
Supplement: Supplementary file 1 [file DataSheet1.docx]

Supplementary Material

## Supplementary Figures


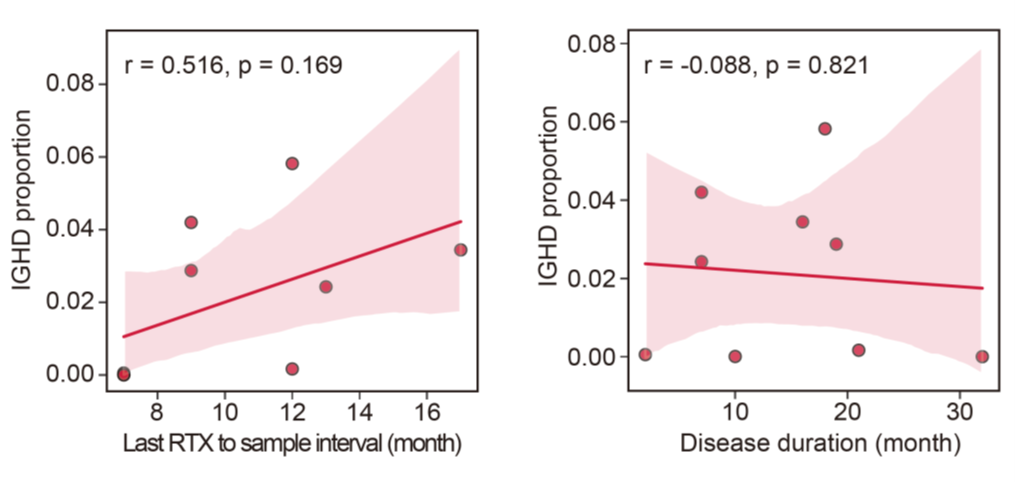


**Supplementary Figure 1.** **Relationship between IGHD proportion and clinical timing in rituximab-treated patients**
Scatter plots show the proportion of IGHD versus (left) interval from the last rituximab (RTX) infusion to sampling and (right) disease duration at sampling (months). Lines indicate simple linear fits with shaded 95% confidence bands. Reported are Pearson’s correlation coefficients (r) with two-sided p-values.
